# DIGITtally—a new tool for streamlining and simplifying *Drosophila melanogaster* meta-analysis

**DOI:** 10.1093/nar/gkaf393

**Published:** 2025-05-13

**Authors:** Andrew Gillen, Shannon Keenan, Maiken Skov, Mehwish Akram, Shireen A Davies, Julian A T Dow

**Affiliations:** School of Molecular Biosciences, University of Glasgow, Glasgow G12 8QQ, United Kingdom; School of Molecular Biosciences, University of Glasgow, Glasgow G12 8QQ, United Kingdom; School of Molecular Biosciences, University of Glasgow, Glasgow G12 8QQ, United Kingdom; School of Molecular Biosciences, University of Glasgow, Glasgow G12 8QQ, United Kingdom; School of Molecular Biosciences, University of Glasgow, Glasgow G12 8QQ, United Kingdom; School of Molecular Biosciences, University of Glasgow, Glasgow G12 8QQ, United Kingdom

## Abstract

*Drosophila melanogaster* has one of the deepest research bases within the life sciences, with a wealth of high-quality tissue- and cell type-specific transcriptomic data available. However, integrating large datasets derived from disparate sources is not trivial. We have designed a broadly applicable solution to this problem in the form of the *Drosophila* Interesting Genes in Individual Tissues-tally (DIGITtally) system. It is freely available online at www.digittally.org. DIGITtally is customizable and hypothesis-free, allowing meta-analysis across the *Drosophila* research space along with analysis of conservation in other species, querying 10 data sources for seven indicators of tissue-specific activity. We have applied DIGITtally to a pertinent question within entomology—that is, whether a specific pattern of gene expression underlies the transporting activity of epithelial tissues (an ‘epitheliome’). By using DIGITtally to survey gene expression throughout the tissues comprising the *D. melanogaster* alimentary canal (salivary gland, midgut, Malpighian tubules, and hindgut), we have verified the existence of a specific ‘epithelial’ vacuolar-type ATPase configuration.

## Graphical Abstract

**Graphical Abstract:**
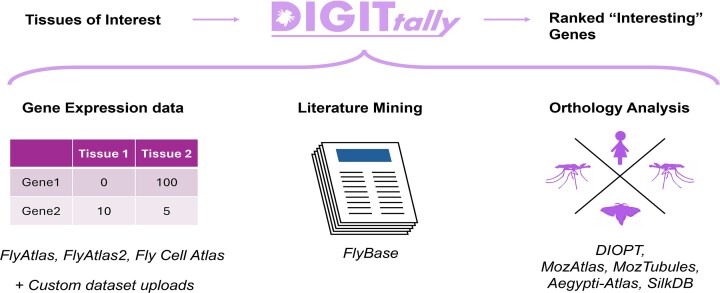

## Introduction

The fruit fly *Drosophila melanogaster* is undoubtedly one of biology’s most studied organisms. The premier fly data repository, FlyBase [1], currently lists over one hundred thousand individual research papers in the field. This is perhaps unsurprising, as fruit fly research not only influences insect research broadly [2, 3], but also has highly translatable implications for human biology [4, 5]. Such a deep research base can enable powerful meta-analyses, though traditionally such approaches are highly technical and time-consuming.

To streamline this process, we have developed a novel approach to automate meta-analysis. This system, termed the *Drosophila* Interesting Genes in Individual Tissues-tally (DIGITtally), is available at www.digittally.org and provides a flexible approach to identifying genes contributing to the function of tissue(s) of interest. DIGITtally takes advantage of information from across the fly research space, utilizing high-profile multi-tissue gene expression datasets, literature searching based on existing text mining data, and orthology information from both humans and select insect species (*Anopheles gambiae*,*Aedes aegypti*, and *Bombyx mori*). Users may also include their own expression data in analyses, further increasing customizability. This website is free and open to all users, and there is no login requirement.

DIGITtally is not limited to analysing genes in the context of a single tissue, but can consider multiple user-defined tissues in combination for genes common to all. Tissue-specific gene expression programs specify cell fate, functionality, and response to stimuli [6–8]. Thus, shared expression pattern may signify shared tissue characteristics. For example, previous work from our group identified that the *Drosophila* transporting epithelial tissues (the midgut, hindgut, salivary gland, and Malpighian tubules) share not only functional similarities, but also an identical pattern of vacuolar-type ATPase (V-ATPase) subunit expression [6, 9].

Here, we describe the rationale underpinning DIGITtally and the various tools provided. To demonstrate the utility of this system, we also provide an illustrative example of the benefits of DIGITtally meta-analysis, focusing on verifying and further identifying gene expression patterns throughout fruit fly epithelia.

## Materials and methods

### DIGITtally implementation

The DIGITtally analysis pipeline was written as a series of open-source Python 3 scripts, while the web implementation utilizes a Django framework. Cookies are used only to manage user data—no cookies are collected for advertising or data aggregation purposes.

Job submission is asynchronous, and many jobs can be run at once. User results are stored for 1 week to allow data retrieval, and then all associated files are purged from the system.

### Data utilization

As of April 2025, DIGITtally utilizes the most recent versions of the following gene-expression datasets: FlyAtlas [10] microarray expression data; FlyAtlas2 [11] RNA-seq expression data; Fly Cell Atlas [12] 10x Genomics ‘Relaxed’ single-nucleus RNA-seq (snRNA-seq) data; MozAtlas [13] expression data; MozTubules [14] microarray enrichment data; Aegypti-Atlas [15] RNA-seq expression data; and SilkDB 3.0 [16] RNA-seq expression data. Insect orthologues of *Drosophila* genes are derived from OrthoDB [17]. Datasets using non-transcriptomic data are also utilized, derived from FlyBase [1] annotation information (gene synonyms, gene to allele linkage, and gene to transcript linkage) and literature-derived fly anatomy ontology information [18] and DIOPT [19] orthology data obtained as a summary from FlyBase. The data underpinning DIGITtally is automatically checked for updates daily, and versions used in each DIGITtally release are listed on DIGITtally’s update pages.

Users may also upload a non-standard RNA sequencing dataset to include in their analysis. In this case, users must upload an expression matrix, such as the output of Salmon [20] or Kallisto [21] pseudo-alignments, along with sample metadata. Examples of these are shown in Supplementary Tables S1 and S2, and also provided on the DIGITtally home page.

### Defining an ‘interesting’ gene

DIGITtally is designed to be applicable to a range of research questions. As such, we have defined various characteristics that outline a gene’s contribution to the behaviour of a tissue set—that is, indicators that a gene is ‘interesting’. These metrics are summed to provide a final by-gene score. The scoring metrics that may be used are defined as:

*Enrichment*: A gene is expressed more highly in chosen tissue(s) than whole insect [7, 22]. In snRNA-seq datasets, this indicates significant enrichment of a gene in target cells compared to all cells (as measured by Bonferroni-corrected Chi-squared test, *P* < .001).

*Specificity*: A gene is expressed most highly in chosen tissue(s) as compared to non-target tissues [6, 23]. In snRNA-seq datasets, specificity is the proportion of cells in target tissue(s) expressing a given gene and/or the inverse of the proportion of non-target cells expressing that gene. It is important to note that specificity is distinct from enrichment (Supplementary Fig. S1).

*Ubiquity*: The proportion of cells belonging to a target tissue that express a gene [24]. This is only usable for snRNA-seq datasets, and differs strongly from specificity (Supplementary Fig. S2).

*Co-expression*: The proportion of cells in an snRNA-seq dataset expressing a given gene that also express any of a user-defined panel of reference genes.

*Published association*: Whether a gene has been linked with target tissue(s) at the transcript, protein, or phenotype level according to FlyBase’s curated anatomy ontology [18], and whether any genetic perturbation of that gene causes a phenotype in a target tissue.

*Human orthology*: Whether a gene has an orthologue in humans and whether that orthologue has a known role in a human disease condition according to OMIM database of human genetic disorders [14].

*Other insect orthology*:A composite score, summarizing whether a gene has an orthologue expressed in homologous tissues in a specified insect and whether that orthologue is enriched and specific to those tissues.

The scores gathered by DIGITtally from each component dataset are summarized in Supplementary Table S3. All metrics may be included to provide final DIGITtally scores for each gene, though none are required, and indeed the user may weight the effects of these scores on the final tally. The equation used to define a gene’s DIGITtally score is defined in Supplementary Fig. S3.

## Results

### The DIGITtally web implementation

The DIGITtally platform contains various user-friendly functions that can be extensively customized, creating outputs tailored to specific research questions (as described below).

#### Analysis wizard

**Figure 1.**
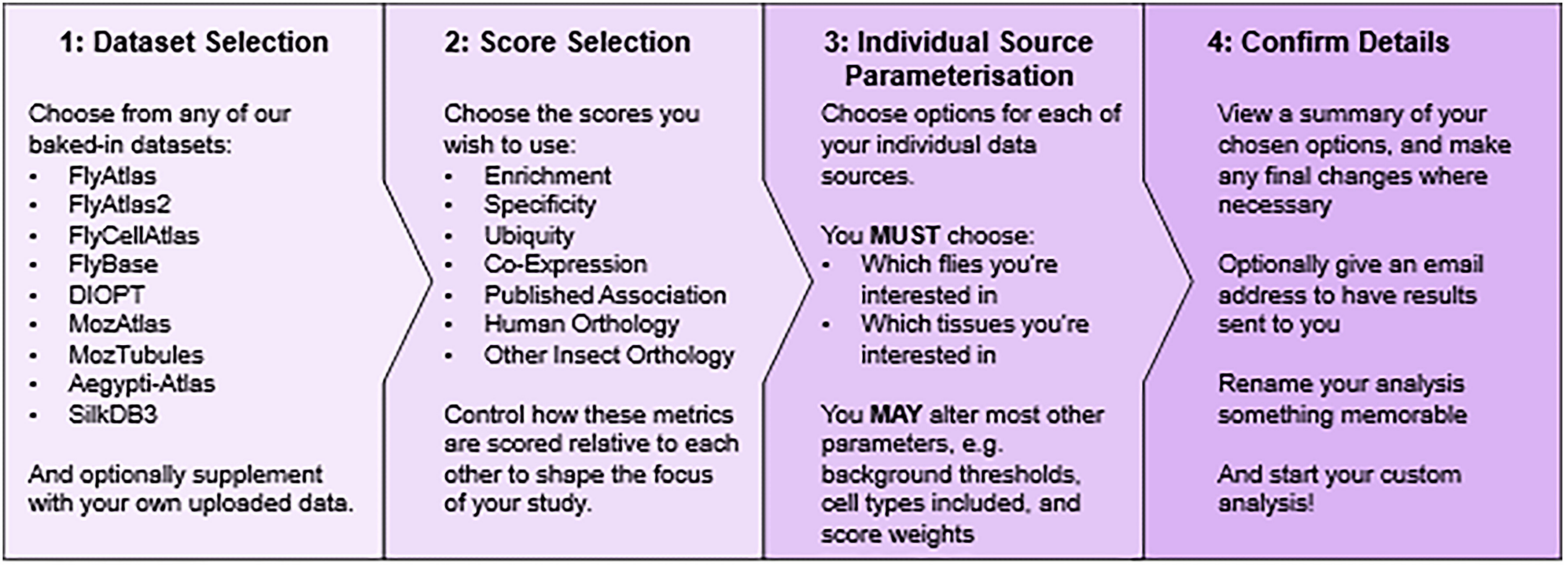
DIGITtally wizard overview. An overview of the process of setting up a new DIGITtally run using our set-up wizard.

This feature is accessible by pressing ‘Begin Wizard’ on any of our pages. Users are guided step by step through a set-up wizard, the structure of which is shown in Fig. 1. Here, users can select any of our 10 data sources and 7 scores (see ‘Materials and methods’ section). Users can also upload their own data to include in our analysis pipeline.

**Figure 2.**
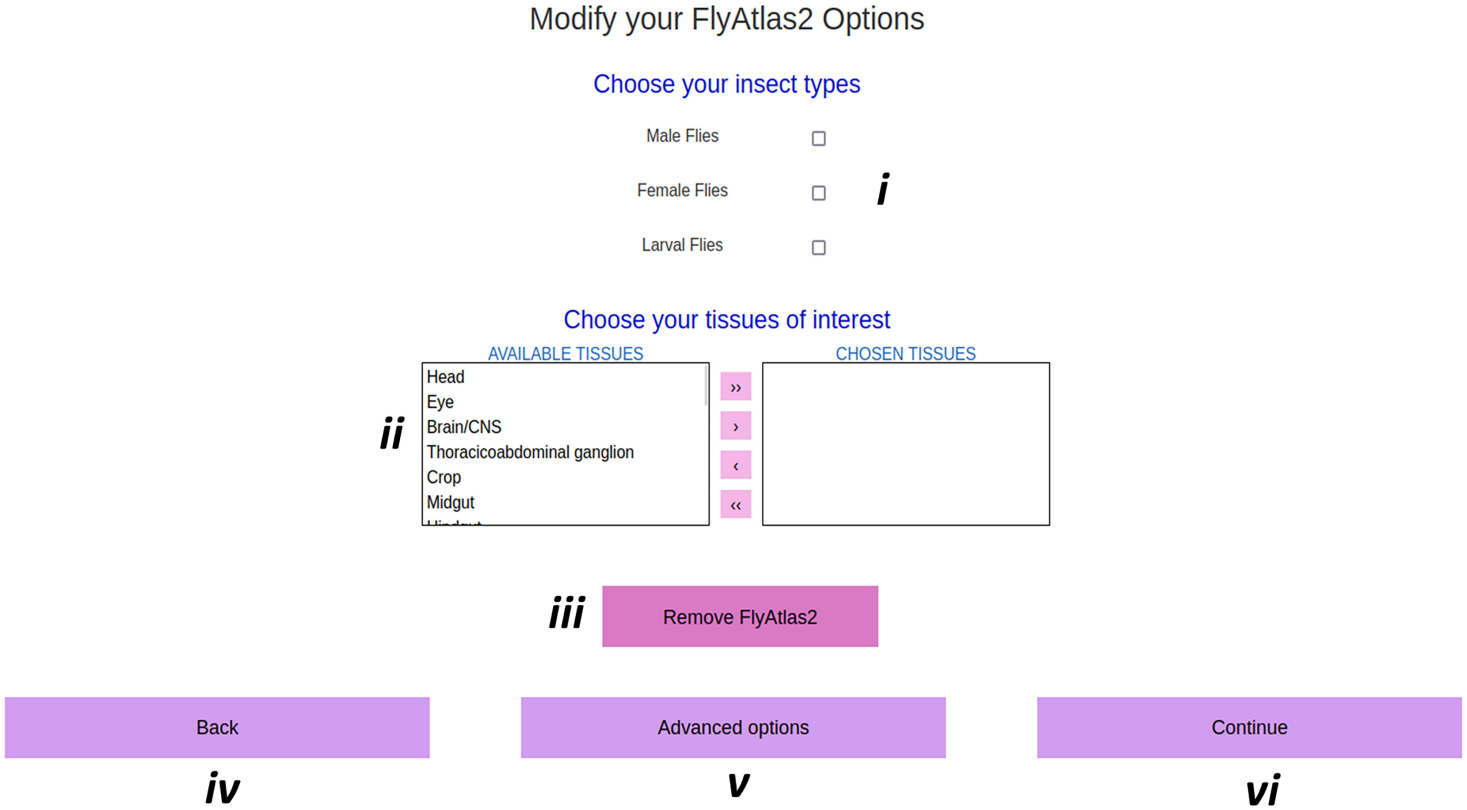
The DIGITtally user interface. The FlyAtlas2 DIGITtally wizard Basic options. The user will choose (**i**) insect types of interest and (**ii**) tissue(s) of interest. (**iii**) Removes the source from analysis without resetting wizard progress. (**iv**) Returns to the previous page. (**v**) Displays advanced options. (**vi**) Continues to the next data source.

This wizard parameterises each source of data in turn, using a simplified yet flexible approach, as shown in Fig. 2. Basic DIGITtally options are limited to the absolute essentials, which users must address to progress. Each page also contains a set of source-specific advanced options that are hidden by default. Once selections have been completed for all datasets in use, users may download their settings as documentation or to upload directly to DIGITtally for replication (see an example in Supplementary Data File S1).

DIGITtally run time will vary depending on analytical complexity, but a simple, single tissue run across all data sources and scores takes ∼30 min. Users can access results directly on the DIGITtally site or via automated email. By default, the top 20 hits from each search are displayed, while complete analytical output can be downloaded on demand.

#### Build a gene list

To ensure user-specified gene lists are recognized by our wizard, e.g. to be used as target genes or reference genes, users may define a set of genes in gene symbol, annotation symbol, or FlyBase ID (FBgn) format. These are converted to DIGITtally-compliant format, with ambiguity flagged for clarification (Supplementary Fig. S4).

Ambiguous identifiers were found by checking all genes for those containing the user input, including one of their known synonyms as listed on FlyBase [1]. This function can be accessed by clicking Other Utilities, then Build a Gene List from the drop-down menu.

#### Pre-built analyses

To provide users with a showcase of DIGITtally’s utility and prevent re-running of basic analyses, DIGITtally maintains a ‘Pre-built Analysis’ facility (Supplementary Fig. S5). This allows users to choose any tissue available in our data, categorized by developmental origin—All, Adult, Male, Female, or Larval—and receive precalculated DIGITtally results. These precalculated runs use all available data and scores for any given tissue, and settings files showing the exact set-up can be downloaded. A full summary of the 60 available pre-built analyses is shown in Supplementary Table S4. This function can be accessed by clicking Other Utilities, then Pre-Built Analyses from the drop-down menu.

### Using DIGITtally to interrogate the *Drosophila* epitheliome

We sought to apply our method to the question of a *Drosophila* epitheliome—that is, whether we can identify genes that are required to define a tissue as ‘epithelial’.

To this end, we carried out a DIGITtally analysis in which midgut, hindgut, Malpighian tubules, and salivary gland were defined as tissues of interest (run settings in Supplementary Data File S1). This analysis used every data source available in our system, along with all scoring metrics barring reference genes, to provide an unbiased analysis. This analysis identified 193 potential epitheliome components, in which higher scores indicate genes that appear most likely to underpin epithelial function.

Table 1 shows the top 20 epitheliome hits (full hits in Supplementary Table S5). Notably, 15 of these top genes encode subunits of V-ATPase (Vha genes). Strikingly, this wholly recapitulates previous findings on epithelial V-ATPase structure [6, 9]. Our meta-analysis also identified several genes, with indicator scores equivalent to those of the V-ATPase subunits, that had not previously been implicated in broad epithelial biology—i.e. the Tetraspanin 29s (Tsp29Fa and Tsp29Fb). Thus, DIGITtally can inform new research directions, as well as verifying previous results. Notably, by changing only a few parameters, this epitheliome search can be tuned to fit other studies, such as identifying novel (unpublished) genes in a given tissue, identifying conserved gene patterns, or finding extremely tissue specific behaviour (Supplementary Fig. S6). In each case, limited parameter tuning radically alters how genes are scored in each case.

**Table 1.** Top hits from the DIGITtally-derived epitheliome

FlyBase ID	Gene symbol	DIGITtally (out of 16)	In Chintapalli *et al.* [6] epitheliome
FBgn0028670	Vha100-2	10.14	Yes
FBgn0027779	VhaSFD	9.69	Yes
FBgn0283535	Vha26	9.58	Yes
FBgn0263598	Vha68-2	9.55	No
FBgn0037671	ATP6AP2	9.55	Yes
FBgn0262512	Vha14-1	9.53	Yes
FBgn0028663	VhaM9.7-b	9.47	Yes
FBgn0262515	VhaAC45	9.34	Yes
FBgn0287825	Vha44	9.32	Yes
FBgn0022097	Vha36-1	9.3	Yes
FBgn0005671	Vha55	9.3	Yes
FBgn0028662	VhaPPA1-1	9.24	Yes
FBgn0285910	VhaAC39-1	9.1	No
FBgn0283536	Vha13	8.76	Yes
FBgn0032075	Tsp29Fb	8.73	Yes
FBgn0262736	Vha16-1	8.46	Yes
FBgn0034365	CG5335	8.42	No
FBgn0000659	fkh	8.06	No
FBgn0262116	RNASEK	7.97	No
FBgn0032074	Tsp29Fa	7.96	No

The top 20 hits returned from the DIGITtally search for genes predicted to have a role across the *D. melanogaster* transporting epithelia (salivary glands, midgut, hindgut, and Malpighian tubules). Genes are listed by their FlyBase ID (FBgn) and current gene symbol, sorted by highest DIGITtally score across all metrics.

## Discussion

We present DIGITtally as a broadly applicable hypothesis generating tool for identifying and ranking genes likely to play a role in a user-defined tissue or set of tissues.

DIGITtally captures the major multi-tissue gene expression datasets that are currently publicly available within insect research, but a modular design ensures that the system can expand to meet the user's needs. For example, if the user has a particular interest in a given tissue, in-depth single-tissue datasets [25, 26] can be included into the meta-analysis in an *ad hoc* fashion. Notably, future development of this resource will seek to include data that are not currently accessible, such as data from the modENCODE project [27], as well as any emerging broadly applicable multi-tissue datasets.

The approach taken by DIGITtally to align genes to a user-defined target ‘pattern’ of characteristics can be compared to the RNA-seq profile tool that FlyBase [1] provides for exploring modENCODE data. As with DIGITtally, users may use this tool to define tissues and life stages in which a target gene would be expressed. Additionally, the modENCODE data can be queried across several experimental perturbations, a feature not possible with the datasets utilized by DIGITtally. That said, DIGITtally represents a meaningful step forward due to not only the inclusion of more than one dataset, increasing the power of its analyses, but also expansion to include gene characteristics outside of raw expression values.

As a gene-finding tool, the output of DIGITtally presents a ready-made gene list that could be used for further analysis using gene set enrichment analysis tools, such as those provided by PANGEA [28] or Gene Ontology [29]. Intuitively, such a strategy would allow users to find processes that are enriched across their tissues of interest.

Tissue- and cell-specific genetics continue to be a key area of interest within fruit fly research [6–8], and DIGITtally is uniquely suited to identifying specific genes with a functional role in tissue determination. Rather than simply interrogating tissue enrichment, the real strength in the system lies in the combination of various types of data from across the research space. Thus, rather than looking for a single ‘smoking gun’ linking genes to tissue(s), DIGITtally builds a comprehensive and comprehensive ‘evidentiary’ case for each gene, ensuring a highly sensitive and holistic approach to hypothesis generation. While DIGITtally is primarily concerned with applying this approach to fruit fly biology, the same strategy could, in the future, be applied to any species of interest.

## Supplementary Material

gkaf393_Supplemental_Files

## Data Availability

DIGITtally is freely available online at www.digittally.org. DIGITtally software is publicly archived through FigShare at 10.6084/m9.figshare.25109450.

## Notes

Present address: Sygnature Discovery, Glasgow G20 0XA, United Kingdom

Present address: SOLASTA Bio, Davidson Building, Glasgow G12 888, United Kingdom
