# Supplementary material for: DIGITtally—a new tool for streamlining and simplifying Drosophila melanogaster meta-analysis
Source: Nucleic Acids Res. 2025 May 13;53(W1):W253–7. doi: 10.1093/nar/gkaf393 (PMC12230737; doi:10.1093/nar/gkaf393)
Supplement: gkaf393_Supplemental_Files [file gkaf393_supplemental_files.zip › DIGITtallyWebserver_SupplementaryData_R3.pdf]

Table Legends

Supplementary Table 1 – DIGITtally Metadata Format. An example of the data format required for user-uploaded metadata. Rows correspond to individual samples, while columns indicate distinct attributes of each data set. Metadata derived from the 2023 update to FlyAtlas2 expression values (see FlyBase reference FBrf0258027, available at http://flybase.org/reports/FBrf0258027.htm.

Supplementary Table 2 – DIGITtally Expression Matrix Format. An example of the data format required for user-uploaded gene expression data. This expression data was generated using Salmon (19) pseudo-alignment. Rows correspond to single genes, while columns indicate individual samples. Expression data corresponds to the 2023 update to FlyAtlas2 expression values (see FlyBase reference FBrf0258027, available at http://flybase.org/reports/FBrf0258027.htm.

Supplementary Table 3 – Scores for each DIGITtally Data Source. Scoring metrics utilised by DIGITtally for each data source implemented. FlyCellAtlas generates two Specificity scores – one for the proportion of cells expressing a gene which are designated as "target" cells – TargetCells; and one for the proportion of non-target cells which do not express the gene – NonTargetCells.

Supplementary Table 4 – Precalculated DIGITtally Runs. A comprehensive account of the precalculated DIGITtally datasets available through the Individual Tissue Tallies utility. For each fly type – Male, Female, Larval, Adult (Male and Female) and All (Male, Female and Larval) – whether or not a DIGITtally run is possible and available for a given tissue is indicated with a tick.

Supplementary Table 5 – Epitheliome DIGITtally Results. All hits from the DIGITtally search of *Drosophila melanogaster* transporting epithelia (midgut, hindgut, Malpighian tubule and salivary glands). A complete breakdown of the scores acquired for each gene for each metric is provided, along with the final DIGITtally score (highlighted).

Supplementary Table 6 – Conserved Enriched Epitheliome DIGITtally Results. All hits from the DIGITtally search of *Drosophila melanogaster* transporting epithelia (midgut, hindgut, Malpighian tubule and salivary glands) when optimised for genes which show strong conservation across all insect species and have a human orthologue (DIGITtally orthology weight set to 3). A complete breakdown of the scores acquired for each gene for each metric is provided, along with the final DIGITtally score (highlighted).

Supplementary Table 7 – Novelty Enriched Epitheliome DIGITtally Results. All hits from the DIGITtally search of *Drosophila melanogaster* transporting epithelia (midgut, hindgut, Malpighian tubule and salivary glands) when optimised for genes which have not been previously studied in this context (DIGITtally Published Association weight set to -10). A complete breakdown of the scores acquired for each gene for each metric is provided, along with the final DIGITtally score (highlighted).

Supplementary Table 8 – Extreme Specificity Epitheliome DIGITtally Results. All hits from the DIGITtally search of *Drosophila melanogaster* transporting epithelia (midgut, hindgut, Malpighian tubule and salivary glands) when optimised for genes which are extremely specific to transporting epithelia (Enrichment thresholds set to require 5-fold higher expression in target tissues over whole fly; and Specificity thresholds set to require 5-fold higher expression in target tissues over non-target tissues). A complete breakdown of the scores acquired for each gene for each metric is provided, along with the final DIGITtally score (highlighted).

Figure Legends

Supplementary Figure 1 - Gene Enrichment versus Specificity in Target Tissues. An illustrative example taken from FlyAtlas2 highlighting genes which achieve DIGITtally scores for **A** – Enrichment, but not Specificity (gene = CG5335) or **B** – Specificity, but not Enrichment (gene = MFS12). In both cases, the target tissues are: Midgut, Hindgut, Malpighian Tubules and Salivary Gland, while Rectal Pad (which sits between these tissues on FlyAtlas2) is marked as non-target for clarity.

Supplementary Figure 2 – Gene Specificity versus Gene Ubiquity. An illustrative example taken from FlyCellAtlas (all cells, 10x Genomics Relaxed data set). Cells which express the gene listed in the panel are coloured black-to-red (based on expression level), while cells which do not express the gene are gray. In both cases, the target cells are from Gut, Malpighian Tubule or Salivary Gland populations (circled). These examples show genes which demonstrate high levels of **A –** Specificity, but not Ubiquity (gene = OtopLb) or **B –** Ubiquity, but not Specificity (gene = scrib).

Supplementary Figure 3 – FlyBase Anatomy Annotation information captures non-transcriptomic publication data. An illustrative example examining the Anatomy ontology terms associated with Tsp29Fa on FlyBase (FB2025_02). **A –** The only anatomical compartment associated with Tsp29Fa in adult *Drosophila* is "head", based on detection at the polypeptide level. This cannot be captured by the bulk transcriptomic data included in FlyBase, as both (**B**) FlyAtlas2 and (**C**) modENCODE datasets find that transcript levels in adult head are negligible. In both cases, transcript expression is given in Reads per Kilobase Million (RPKM), as reported by FlyBase.

Supplementary Figure 4 – The DIGITtally Calculation. The full equation used to calculate the DIGITtally score for each gene. Each individual source (indicated with a different colour for clarity) may be weighted by the user individually – the weight outside the parentheses. Each individual scoring metric can also be weighted globally (indicated by "gw", Global Weight, within the equation; and separately for the individual source, as indicated by "sw", Source Weight, within the equation, coloured to indicate the corresponding source. All

Orthology measures also share a common Orthology ("Ortho") Weighting. By default, all weights are set to 1 indicating equivalent weighting. The only exception is the MozTubules Weight, which is 0.3 by default, as only Enrichment can be analysed in MozTubules data.

Supplementary Figure 5 – The DIGITtally Genelist Builder. A – The Gene Entry page for the Genelist builder utility. For this example, a user has entered CG5335 (a unique, real identifier); NOT_A_GENE (an unrecognized identifier); and P1 (an ambiguous identifier). **B –** The disambiguation page. Based on user inputs, they may be informed that a gene cannot be recognised, as for NOT_A_GENE. Alternately, if an identifier could refer to multiple genes, they must choose one of the possibilities, as for P1 in this example. They may then receive a file containing FBgn identifiers for each of their genes.

Supplementary Figure 6 – DIGITtally Single Tissue Selector. A – The Tissue Selection page for the Individual Tissues Tally utility. For this example, a user has elected to receive results based on a DIGITtally search for genes which look "interesting" in Malpighian Tubules of all fly types (Male, Female and Larval). The "Get Results" page redirects to the next screen. **B –** The Individual Tissues results page. The top 20 genes based on their DIGITtally score are displayed in a results table. From this page, users can download the settings used to generate these results, along with the full breakdown of the top 250 DIGITtally-ranked genes from the tissue set (in a .csv); and the full file set associated with the DIGITtally run (in a .zip folder).

Supplementary Figure 7 – DIGITtally parameterisation drastically affects result ordering. The Epitheliome run settings (**Supplementary Data File 1**) were modified to represent three alternate use cases: Enriching for highly-conserved epitheliome components (Conserved Enriched); Suppression of hits which have previously been studied (Novelty Enriched); and Limiting scores to be more selective for extremely tissue-specific behaviours (Extreme Specificity). The settings altered in each case are shown, along with the top ten DIGITtally hits, illustrating how changing parameters can control which genes of interest are returned as the "best hits". Full tables for these runs are provided in **Supplementary Tables 6-8.**

Supplementary Figures

Supplementary Figure 1

A

Tissue	Adult Male FPKM	Adult Male Enrichment
Head	5.8	0.5
Eye	7.6	0.6
Brain / CNS	2.2	0.2
Thoracicoabdominal ganglion	5.0	0.4
Crop	35	3.0
Midgut	68	5.7
Hindgut	37	3.1
Malpighian Tubules	38	3.2
X Rectal pad	36	3.1
Salivary gland	43	3.6
Fat body	11	0.9
Heart	16	1.3
Trachea		
Ovary		
Virgin Spermatheca		
Mated Spermatheca		
Testis	3.5	0.3
Accessory glands	39	3.3
Carcass	13	1.1
Garland cells		
Whole body	12	

Target Tissues (Midgut, Hindgut, Malpighian Tubules, Rectal pad, Salivary gland)

All target tissues show enrichment over whole body

Non-target tissue shows higher expression than some target tissues

B

Tissue	Adult Male FPKM	Adult Male Enrichment
Head	0.2	0.1
Eye	0.4	0.1
Brain / CNS	0.8	0.2
Thoracicoabdominal ganglion	1.9	0.4
Crop	0.4	0.1
Midgut	28	5.7
Hindgut	20	4.1
Malpighian Tubules	105	22
X Rectal pad	0.1	0.0
Salivary gland	5.2	1.1
Fat body	0.2	0.0
Heart	1.1	0.2
Trachea		
Ovary		
Virgin Spermatheca		
Mated Spermatheca		
Testis	1.7	0.3
Accessory glands	0.4	0.1
Carcass	1.4	0.3
Garland cells		
Whole body	4.9	

Target Tissues (Midgut, Hindgut, Malpighian Tubules, Rectal pad, Salivary gland)

Not all target tissues show notable enrichment over whole body

Expression in all non-target tissues is notably lower than any target tissue

Supplementary Figure 2

A

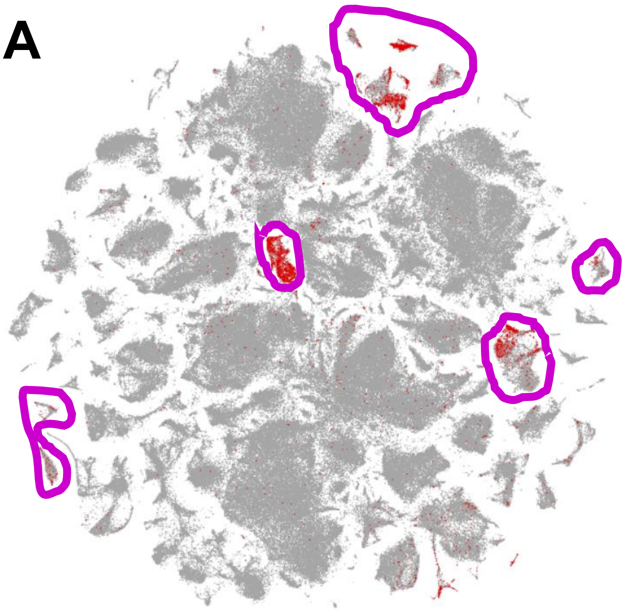

OtopLb

Moderate to High Specificity – 60% cells expressing gene are Target cells.

Low Ubiquity – 18% of Target cells express the gene.

B

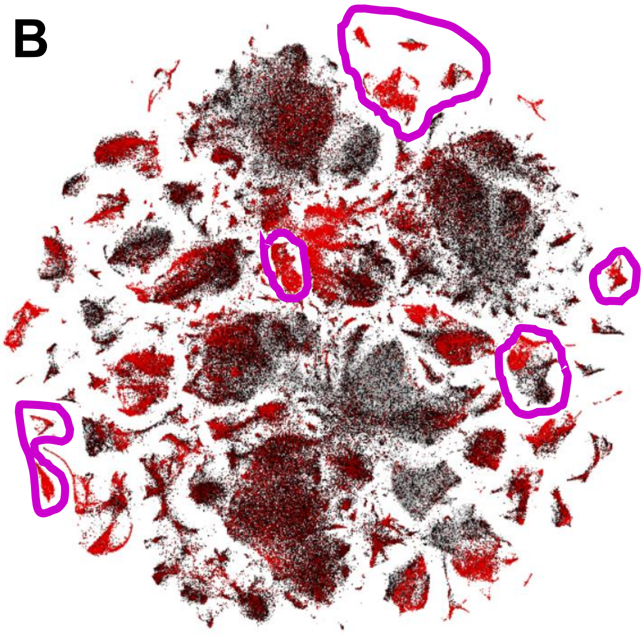

scrib

Very Low Specificity – <1% cells expressing gene are Target cells.

High Ubiquity – 67% of Target cells express the gene.

Supplementary Figure 3

A

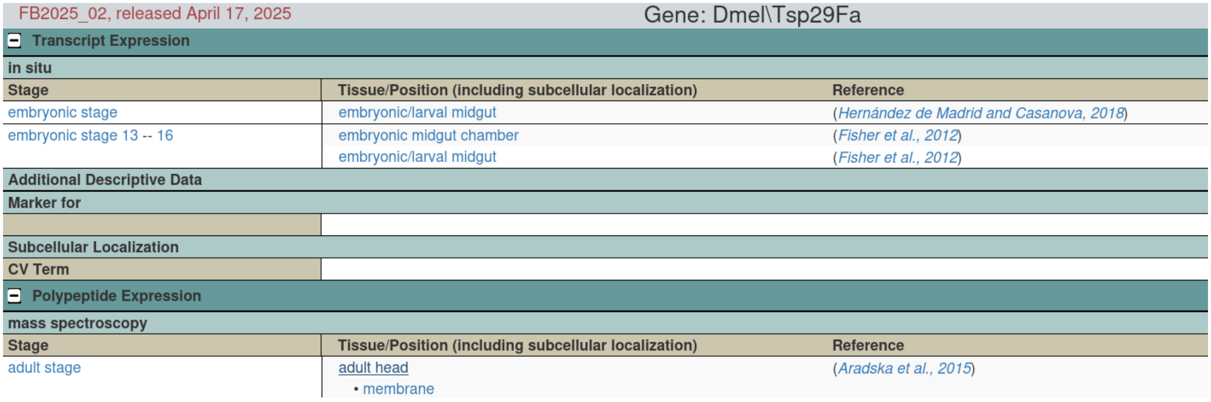

FB2025_02, released April 17, 2025 — Gene: Dmel\Tsp29Fa

Transcript Expression

in situ

Stage	Tissue/Position (including subcellular localization)	Reference
embryonic stage	embryonic/larval midgut	(Hernández de Madrid and Casanova, 2018)
embryonic stage 13 -- 16	embryonic midgut chamber	(Fisher et al., 2012)
	embryonic/larval midgut	(Fisher et al., 2012)

Additional Descriptive Data

Marker for

Subcellular Localization

CV Term

Polypeptide Expression

mass spectroscopy

Stage	Tissue/Position (including subcellular localization)	Reference
adult stage	adult head<br>• membrane	(Aradska et al., 2015)

B

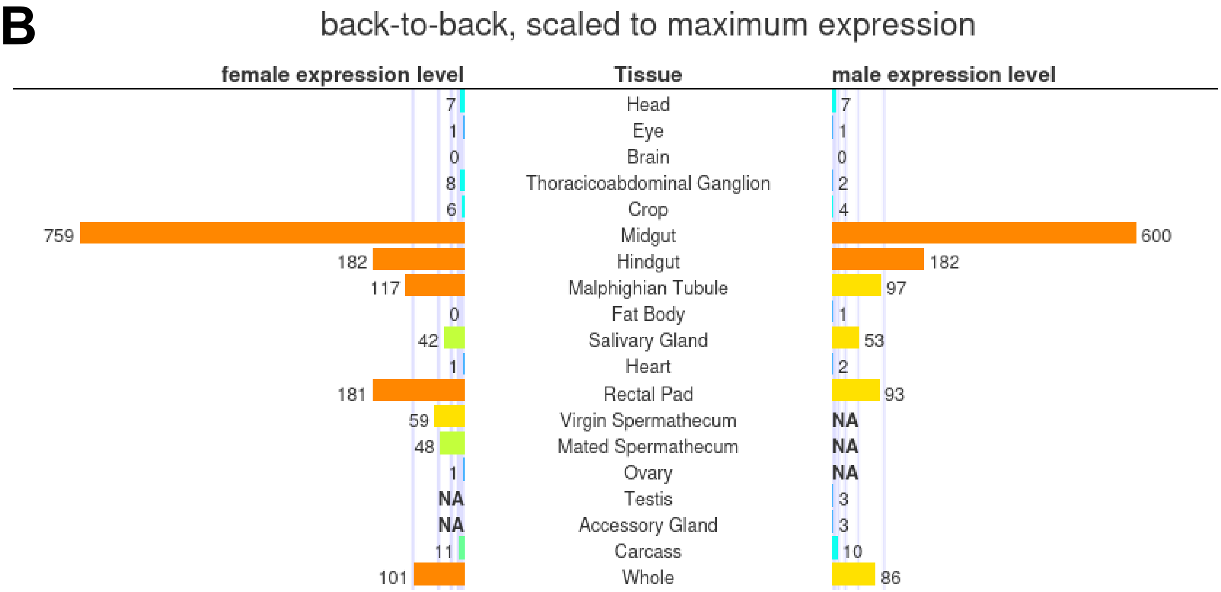

C

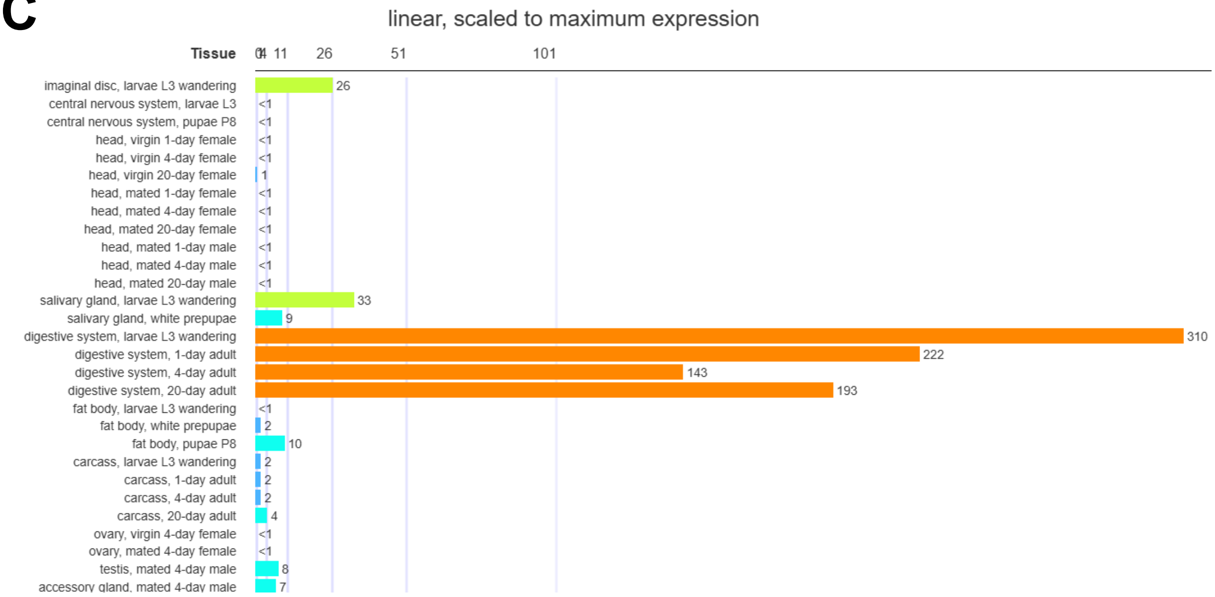

Supplementary Figure 4

FlyAtlas ▶ $FlyAtlas1\ Weight \times [(gw \times sw \times Enrichment) + (gw \times sw \times Specificity)]$

\+ FlyAtlas2 ▶ $FlyAtlas1\ Weight \times [(gw \times sw \times Enrichment) + (gw \times sw \times Specificity)]$

\+ FlyCellAtlas ▶ $$FlyCellAtlas\ Weight \times \begin{bmatrix} (gw \times sw \times Enrichment) \\ + (gw \times sw \times Specificity(TargetCells)) \\ + (gw \times sw \times Specificity(NonTargetCells)) \\ + (gw \times sw \times Ubiquity) \\ + (gw \times sw \times CoExpression) \end{bmatrix}$$

\+ FlyBase ▶ $$FlyBase\ Weight \times \begin{bmatrix} (gw \times sw \times Any\ Association\ with\ Tissue) \\ + (gw \times sw \times Mutant\ Phenotype\ in\ Tissue) \end{bmatrix}$$

\+ *Homo sapiens* ▶ $$\begin{bmatrix} H.sap\ Weight \\ \times Ortho\ Weight \end{bmatrix} \times \frac{\begin{bmatrix} (sw \times Any\ Human\ Ortholog) \\ + (sw \times Disease\ Associated) \end{bmatrix}}{2}$$

\+ *Anopheles gambiae* ▶ $$\begin{bmatrix} A.gam\ Weight \\ \times Ortho\ Weight \end{bmatrix} \times \frac{\begin{bmatrix} (sw \times Enrichment) + (sw \times Specificity) \\ + (sw \times Expression) \\ + (Moztubules\ Weight \times Moztubules\ Enrichment) \end{bmatrix}}{3 + Moztubules\ Weight}$$

\+ *Aedes aegypti* ▶ $$\begin{bmatrix} A.aeg\ Weight \\ \times Ortho\ Weight \end{bmatrix} \times \frac{\begin{bmatrix} (sw \times Enrichment) + (sw \times Specificity) \\ + (sw \times Expression) \end{bmatrix}}{3}$$

\+ *Bombyx mori* ▶ $$\begin{bmatrix} A.aeg\ Weight \\ \times Ortho\ Weight \end{bmatrix} \times \frac{\begin{bmatrix} (sw \times Enrichment) + (sw \times Specificity) \\ + (sw \times Expression) \end{bmatrix}}{3}$$

\+ User Data $User\ Data\ Weight \times [(gw \times sw \times Enrichment) + (gw \times sw \times Specificity)]$

→ **TOTAL DIGITtally Score**

Supplementary Figure 5

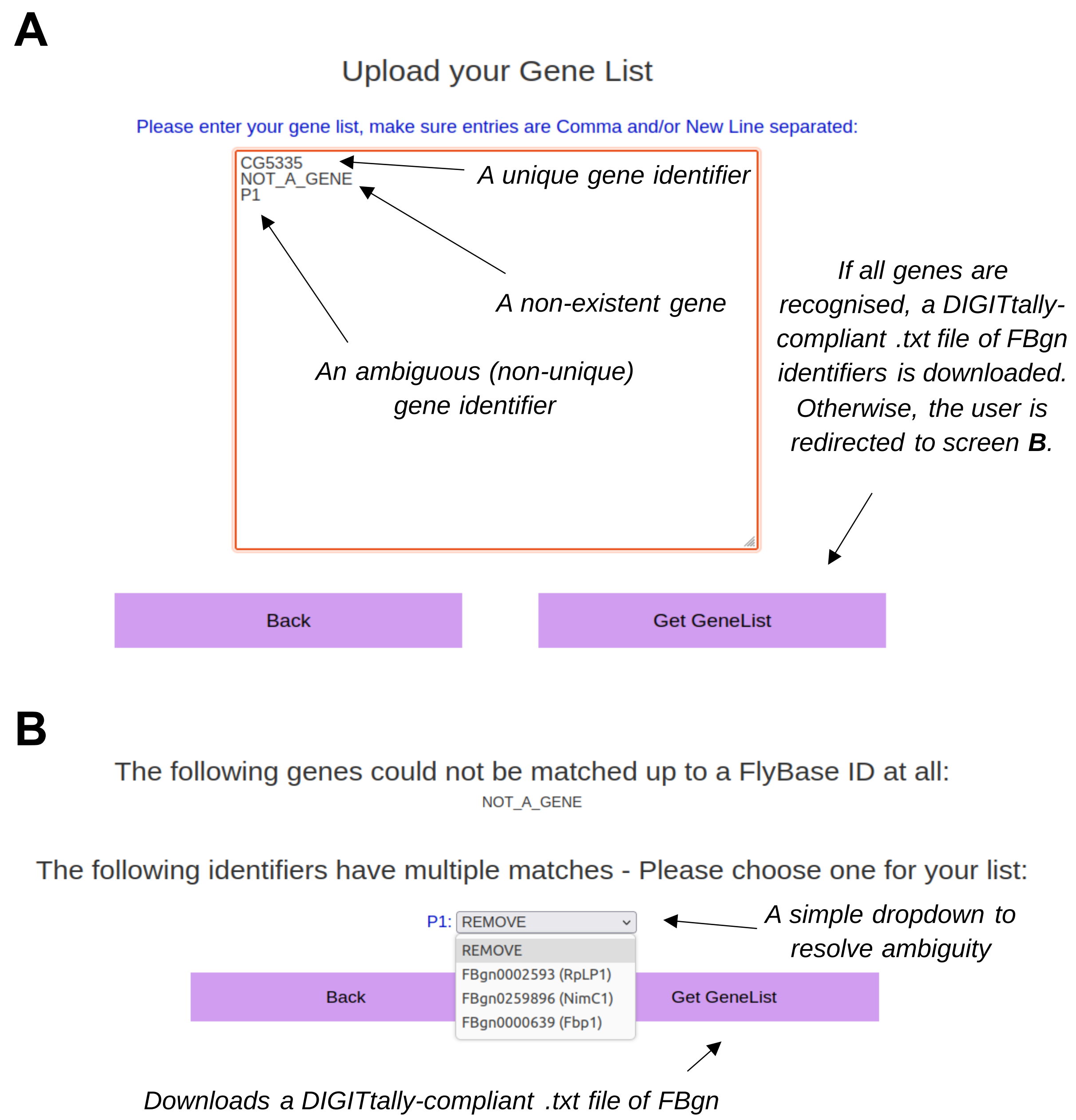

Supplementary Figure 6

A

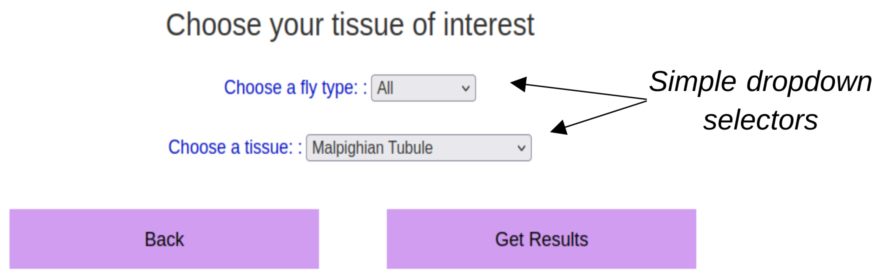

B

Below are the top twenty DIGITtally identified results for Malpighian Tubule in ALL flies, ranked by their total HitScore.

To see further Hits, or to see a full breakdown of the DIGITtally calculation, you can use the "Download Full DIGITtally Sheet" button at the bottom of the page

You can also get the FULL package of files used to generate this score using the "Download ALL DIGITtally files" button

If you want to check the settings for this run, the settings file can be downloaded
HERE

The run's settings can be downloaded as a .txt file

FlyBase ID	Gene Symbol	DIGITtally (out of 14 MAXIMUM)
FBgn0004198	ct	12.588568634911368
FBgn0034715	Oatp58Db	11.780102556537818
FBgn0029896	CG3168	11.323345104833745
FBgn0039872	salt	11.281632345191504
FBgn0032706	Irk3	11.232948988300162
FBgn0039519	Cyp6a18	11.170475118692497

The top 20 genes are shown in tabular form, while full DIGITtally output can be downloaded for local use

Supplementary Figure 7

All settings and weights as default	Orthology score weight increased from 1 to 3	"Any Association" Published Association reduced to -10	Enrichment and Specificity Thresholds set to 5
**BASE settings**	**Converved Enriched**	**Novelty Enriched**	**Extreme Specificity**
Vha100-2	Cyp9c1	Tsp29Fb	Vha26
Vha26	Vha100-2	CG5335	Vha55
ATP6AP2	Tsp29Fb	RNASEK	Vha68-2
Vha68-2	Cyp12a4	Cyp9c1	VhaSFD
VhaSFD	Tsp29Fa	CG46491	Vha16-1
VhaAC45	by	Fnta	Pdhb
Vha55	Cyp12e1	α-Est3	OtopLb
Vha14-1	Vha26	Vha14-1	Ald1
VhaM9.7-b	Vha13	CG7255	Rpl7A
VhaAC39-1	α-Est3	CG31751	mtd

Additional Supplementary Data

Supplementary Data File 1 – DIGITtally Settings file used to run the Epitheliome search
